# Antibacterial Activities of Selected Plant Extracts on Cariogenic Microbes

**DOI:** 10.3290/j.ohpd.c_2851

**Published:** 2026-11-11

**Authors:** Amal Sabour, Alanoud Al-Rajeh, Shada Al-Anazi, Horiah A. Aldehaish, Girija A. S. Smiline, Shoba Gunasekarand, Nadine M.S. Moubayed

**Affiliations:** a Amal Sabour Professor, Department of Botany and Microbiology, College of Science, Female Campus, King Saud University, Riyadh, Saudi Arabia. Supervision, edited the manuscript, read and agreed on the final manuscript.; b Alanoud Al-Rajeh Lecturer, Department of Botany and Microbiology, College of Science, Female Campus, King Saud University, Riyadh, Saudi Arabia. Conceptualization, experimentation, read and agreed on the final manuscript.; c Shada Al-Anazi Lecturer, Department of Botany and Microbiology, College of Science, Female Campus, King Saud University, Riyadh, Saudi Arabia. Conceptualization, molecular docking analysis, experimentation, read and agreed on the final manuscript.; d Horiah A. Aldehaish Researcher, Department of Botany and Microbiology, College of Science, Female Campus, King Saud University, Riyadh, Saudi Arabia. Experimentation, read and agreed on the final manuscript.; e Girija A. S. Smiline Professor, PhD student, chemist, lecturer etc. NOT academic degree Department of Microbiology, Saveetha dental college and hospitals, Saveetha Institute of Medical and technical sciences[sIMats], Saveetha University, Chennai, Tamilnadu, India. Edited the molecular docking analysis, read and agreed on the final manuscript.; f Shoba Gunasekarand Chemist and Lecturer, Department of Biotechnology, Dwaraka doss Goverdhan doss Vaishnav College (autonomous), University of Madras, Chennai, Tamil Nadu, India. Performed molecular docking experiments, read and agreed on the final manuscript.; g Nadine M.S. Moubayed Researcher, Department of Botany and Microbiology, College of Science, Female Campus, King Saud University, Riyadh, Saudi Arabia. Wrote and edited the first draft.

**Keywords:** derum, charcoal, miswak, molecular docking, mouthwash, sodium bicarbonate, turmeric.

## Abstract

**Purpose:**

To investigate and compare the antibacterial effect of different botanical (turmeric, miswak, derum) and non-botanical oral hygiene products (toothpaste, mouthwash, activated charcoal, baking soda) on the cariogenic bacteria *E. coli, S. aureus*, and *S. mutans. *

**Materials and Methods:**

Different plant extracts (turmeric, miswak, and derum) and as well as non-botanical substances used in oral hygiene (charcoal, toothpaste, mouthwash, and sodium bicarbonate) were studied for their antibacterial activities against cariogenic model microbes *Streptococcus mutans, Staphylococcus aureus*, and *Escherichia coli* using the agar diffusion technique and in-silico molecular docking. Based on LC-MS analysis and literature reviews, linoleic acid – the bioactive fatty acid antimicrobial and anti-biofilm agent present in the studied plants – was selected for molecular docking analysis.

**Results:**

Among all tested materials, derum revealed the highest activity against *S. mutans* (inhibition zone diameter 16 mm^2^), *S. aureus* (inhibition zone diameter 14.3 mm^2^), and *E. coli* (inhibition zone diameter 14 mm^2^). The mouthwash and toothpaste inhibited the growth of *S. aureus* (inhibition zone diameter 15 mm^2^) and *E. coli* (14 mm^2^) but had no or negligible effect on *S. mutans* (8 mm^2^) as was the case for all the other substances used in oral hygiene tested here. Liquid chromatography/mass spectrometry revealed the presence of linoleic acid at 279m/z, and in-silico screening demonstrated a possible affinity of linoleic acid for extracellular polysaccharide glucan. Low energy level scores of -1.24 kcal/mol stabilized by four hydrogen bonds between linoleic acid and extracellular alpha (α) 1,3-glucan matrix showed promising potential of linoleic acid in the treatment of *S. mutans* biofilm.

**Conclusion:**

Derum sticks showed the highest antibacterial activity against *S. mutans*, followed by *S. aureus*, and *E. coli*. The insights from molecular docking analysis of linoleic acid and glucan may lead to the development of therapeutic agents targeting extracellular polymeric substance (EPS) synthesis, thus helping prevent biofilm formation and improve oral hygiene.

Dental caries is one of the world’s most common diseases, although it is rarely life-threatening; however, it is a major concern for healthcare providers. A deep understanding of the microorganisms’ role in dental diseases is required to hinder caries formation. A biofilm, defined as a complex layer of bacterial cells, saliva or saliva substitutes (e.g. lubricating gels or sprays), and food residue, attaches to tooth surfaces and is embedded in a matrix of extracellular polymeric substances (EPS), leading to a change in the properties of microorganisms and increasing resistance to antimicrobial agents. Uncontrolled, this biofilm on the tooth surfaces can easily reach a thickness of hundreds of cells. The developed biofilm, also known as plaque, provides an ideal environment for many bacterial species to colonize and grow.^12^ More than 700 bacterial taxa have been found in the oral cavity, most of these microbes are harmless, but some may cause diseases such as caries or periodontitis under certain circumstances.^32^

*Streptococcus mutans* (*S. mutans*) is the most important cariogenic microbe, and it depends on different virulence factors – such as osmolarity, pH, and carbon source availability – for the production of a strong biofilm.^12^ *S. mutans* can adhere to the tooth surface through high-affinity adhesins. Moreover, it has high sucrose consumption which leads to high acid production and hence high acid tolerance as well as the ability to grow at lower pH.^6^ This mature biofilm plays a critical role in caries and plaque formation.^20^ Furthermore, *S. mutans* synthetizes EPS by the formation of water-insoluble glucans (glucosyltransferases and fructosyltransferase as well as beta-1,3 glucan), increasing adhesion stability and integrity of the biofilm on the tooth surface; EPS acts as a scaffold for other oral pathogens to colonize and form dental plaque.^19^

Diverse manufacturer products such as CHX and other non-botanical synthetic antibacterial substances have been used either to reduce oral biofilms or inhibit bacterial strains associated with specific oral diseases. Meanwhile, several studies have demonstrated that bacterial antibiotic resistance is on the rise in the oral cavity.^7,27^ Due to the reduced efficacy of synthetic agents, there is a growing emphasis on using plant-based alternatives to control oral biofilms.^16^ Plant-based substances, including polyphenols, flavonoids, terpenoids, and linoleic acid (plant fatty acid) have been found to reduce the bacterial count of adherent microorganisms. They all exhibit anti-adhesive and antimicrobial activity based on different mechanisms. For example, linoleic acid has been reported to exhibit antibacterial activity particularly against Gram-positive bacteria, by disrupting the bacterial cell membranes, leading to cell lysis.^9,14^ Linoleic acid has been studied for its ability to inhibit the production of extracellular polysaccharides (EPS) by *Streptococcus mutans*.^1^ Similarly, cranberry extract has strong anti-adhesive properties and pronounced biofilm inhibition thanks to reducing bacterial membrane hydrophobicity.^16^ Black, green and some herbal tea extracts are also thought to cause bacterial death due to bacterial cell-membrane lysis.^29^

Certain plant-derived substances have long been used in specific geographical regions. Derum, for instance, is a natural “chewing stick” obtained from the bark of the walnut tree (*Juglans regia* L.) and is widely appreciated for its antimicrobial, antioxidant, hypoglycaemic, and antihypertensive properties, in addition to the use of its dried bark as a cleaner and whitener for teeth.^15^ Derum can reduce caries and gum disease when used as a chewing stick, since it improves mechanical and chemical cleaning of teeth and oral tissues compared to a standard toothbrush.^3^ Turmeric (*Curcuma longa*) is another example of a plant product commonly used in oriental medicine for the treatment of toothache, gastritis, vomiting, jaundice, and contusions.^21^ Miswak (*Salvadora persica*) sticks, the precursor to the modern-day toothbrush, were used in Europe until about 300 years ago,^23^ and are still used today in many countries in Asia, Africa, and the Middle East for oral hygiene, also in toothpick form.^4^ In addition to its antimicrobial activity,^2^ it possesses a high content of trimethyl amine, chlorides, high levels of fluoride and silica, sulfur, vitamin C, small amounts of tannins, saponins, flavonoids, and sterols,^21^ which are important elements for remineralizing dental enamel.

On the other hand, different chemical compounds have exhibited similar effects on oral hygiene. Charcoal, or activated carbon, is one of the widely used inorganic compounds in toothpaste preparations for the removel of stains and plaque. It absorbs large amounts of noxious substances, including toxic exudates from diseased gums.^13^ However, charcoal’s structure, composition, and particle sizes increase its abrasive properties, and hence roughen the surface of dental enamel.

Another chemical product widely used in controlling caries/protecting the teeth is toothpaste. Various synthetic or natural antimicrobial agents have been used in toothpastes,^28^ all serving the purpose of reducing bacterial count, preventing dental biofilm formation, and in the case of fluoride toothpaste, remineralizing dental hard tissues. Mouthwash is also used for preventing oral diseases. Particularly, mouthwash containing an antiseptic – mainly chlorhexidine – is considered the gold standard in reducing dental plaque and pathogenic microorganisms, including *S. mutans*. Currently, herbal mouthwashes containing miswak are instead being used increasingly extensively, indicating the return to phyto-alternatives for the control and treatment of oral diseases. Sodium bicarbonate or baking soda is also known for controlling and inhibiting *S. mutans* growth and is commonly used in non-abrasive toothpastes. An additional factor in favor of its use in dental hygiene is that it is easily available.^25^

The present study aimed to investigate and compare the effect of different plant extracts and non-botanical substances on controlling oral hygiene. Turmeric, miswak, derum, toothpaste, mouthwash, charcoal, and baking soda were tested for their antibacterial activities and their influence on the survival and diversity of *E. coli, S. aureus*, and *S. mutans* was determined using the agar diffusion technique. Thus, to facilitate faster transition to clinical situations, the following are necessary: more comprehensive knowledge of bio-molecular interactions and designing and re-designing chemical molecules using advanced computer technology, in order to reveal the structural chemical properties and enhance an understanding of the inhibition mechanisms.^8^ In this context, in-silico molecular docking was applied to assess the interaction and recognition between linoleic acid – a phyto-constituent revealed by LC-MS at m/z 279,^17^ which is present in derum,^16^ and used here as the ligand – and the extracellular glucan polymer abundant in *S. mutans* biofilm. By predicting these possible interactions between linoleic acid (LA) and alpha (α)1,3-glucan oligosaccharide model, a newly structured drug might be developed, and new, plant-based therapeutic agents could be screened targeting specific virulence factors in bacterial biofilm formation.^19^

## MATERIALS AND METHODS

### Sample Collection and Preparation

All sampleswere purchased from a local market in Riyadh, Saudi Arabia. The plant samples were further identified at the Botany Department of King Saud University. *Juglans regia* L. (walnut) bark (derum), turmeric powder derived from the rhizome of *Curcuma longa*, and miswak sticks (*Salvadora persica* root) were pulverized and 25 g of the obtained powder was added to 50 ml of sterile distilled water, then incubated in a rotary shaker for 3 days at room temperature. Simultaneously, 5 g of each of Oralgin Charcoal Toothpaste (Dr. Sulaiman Al Habib Medical Group; Riyadh, Saudi Arabia), Aloedent Anti-Cavity Sensitive toothpaste (Optima Naturals; Vergiate, Italy), both fluoride-free, Colgate mouthwash (Colgate Palmolive; New York, NY, USA), and sodium bicarbonate were placed directly in 10 ml of sterile distilled water and shaken as above.

### Liquid Chromatograph/Mass Spectrometer (LC-MS/MS) Analysis

Chromatographic grade ethanol, ammonium acetate, and acetonitrile (Sigma Aldrich; St Louis, MO, USA) were used. Milli-Q ultrapure deionized water was used in all experiments (Millipore; Burlington, MA, USA). The liquid chromatograph mass spectrometer, 6460, Triple Quad LC-MS (Agilent Technologies; Santa Clara, CA, USA) was equipped with an Agilent Eclipse XDB C18 column (100 x 2.1 mm^2^).

Mass spectrometric detection was carried out in electrospray ionization (ESI) negative-ion mode. Nitrogen was used as the drying and sheath gas at a flow rate of 13 l/min at 250°C. The mobile phase consisted of A: 2 mm ammonium acetate in water and B: acetonitrile. The column temperature was maintained at 30°C, the sample volume was prepared in 1 ppm methanol, and 10 μl of the derum extract was injected at a flow rate of 0.35 ml/min. The gradient profile was set as follows: 50% A (0-16 min), 100% B (16-20 min), 50% B (20-25 min).

### Bacterial Inoculum Preparation

Three bacterial species were tested: *Staphylococcus aureus, Escherichia coli,* and *Streptococcus mutans*. These clinical isolates were provided by the Microbiology Department, King Khaled University Hospital. Pure strains were pre-cultured on nutrient agar plates before each experiment, and then fresh bacterial suspensions of 0.5 MacFarland were prepared in 5 ml sterile distilled water.

### Antibacterial Activity Assessment 

The antibacterial activity of the botanical extracts and the non-botanical products was determined using the agar-well diffusion technique on Muller Hinton agar plates (MH) (Oxoid; Lenexa, KS, USA). Briefly, an inoculum of each of the prepared bacterial suspensions of *S. aureus, E. coli,* and *S. mutans* was placed on the surface of MH agar plates with a sterile cotton swab. Then, wells were punched into the agar with a sterile metal cylinder (8 mm diameter), and filled with 200 µl of each sample extract (one sample for each plate). The plates were incubated at 37°C for 24 h. The plates were observed for clear inhibition zones around the wells, and the diameter of the inhibition zone (DIZ) was measured using a ruler. The mean diameter of the inhibition zones around each well was calculated. Each sample was tested three times.^2^ Data were compared to the standard antibiotic disk of erythromycin (15 µg) as a positive control.

### Statistical Analysis

Descriptive statistical analysis was performed, and the results were expressed as mean ± standard deviation, with median values also calculated where appropriate. Differences between groups were evaluated using Student’s t-test, and a p-value < 0.05 was considered statistically significant.

### Retrieval of Alpha 1,3-glucan and Linoleic Acid Structure

Chemsketch software (ACD Labs; Toronto, Ontario, Canada) was used to observe linoleic acid’s structural configuration, and to generate and draw its 3D structure. The selected ligand was saved as a MOL file, converted by an open-label molecular converter program and saved as a PDB file.^30^

Similarly, alpha (α)1,3-glucan was selected since it is abundant in bacterial biofilm. Its structure was retrieved using a Biovia Discovery studio visualizer (Dassault Systèmes; San Diego, CA, USA).

### Molinspiration Assessment for Drug Likeliness

The Molinspiration assessment program (Molinspiration; Bratislava, Slovakia) was used for determining the linoleic acid molecular descriptors including logP for partition co-efficient, the compounds’ molecular weights, hydrogen bond acceptors and donors counts for their membrane permeability and bioavailability.^29,31^ Further assessments on absorption, distribution, metabolism, and elimination (ADME) of the ligands were evaluated by Lipinski rule of five.^22^

### Molecular Docking Analysis: Affinity Between Linoleic Acid and Beta-Glucan

The affinity between linoleic acid the alpha-glucan polysaccharide model was interpreted using the AutoDock 4.2 tool (La Jolla, CA, USA).^24^ Using an auxiliary Autogrid program (AutoGrid Systems; Redwood City, CA, USA), alpha 1,3-glucan was embedded in preset grid maps, one each assigned to the type of atoms present in the compound being docked.^24^ The 12–10 and 12–6 parameters given by Lennard-Jones^24^ were applied to model all H–bonds and van der Waals forces, respectively. The Discovery Studio Visualizer was used to determine the hydrogen bonds between linoleic acid and alpha-glucan. Their molecular interactions for binding were assessed for their relative stabilities.

## RESULTS

Data from the present study revealed that turmeric, miswak, charcoal, and sodium bicarbonate did not affect the tested bacteria, as no inhibition zones were observed around the corresponding wells. In contrast, the toothpaste, mouthwash, and derum were all effective against *E. coli, S. aureus*, and *S. mutans*. The highest activity was observed with derum extract on *S. mutans* (16±0 mm^2^), followed by the Gram-negative *E. coli* and the Gram-positive *S. aureus*, each with a DIZ of 14±0 mm^2^.

The mouthwash, on the other hand, exhibited higher activity on *S. aureus* (14±0.57 mm^2^), followed by *E. coli* (12±0.76 mm^2^) but had a negligible effect on *S. mutans* (8 mm^2^). The activity of the Aloedent Anti-Cavity Sensitive toothpaste resembled that of the mouthwash on *S. aureus*, with a DIZ (14±0 mm^2^) and had negligible effect on both strains *S. mutans* and *E. coli *(8 mm^2^). These results were compared to the standard antibiotic erythromycin as indicated in Table 1. The p-value was >0.05 (Student’s t-test), indicating that there was no statistically significant difference in the antibacterial activities of the extracts against the tested bacterial strains. The highest activity was observed for derum on *S. mutans* (Fig 1).

**Table 1:** Antibacterial activity of the various plant and non-botanical substances used in oral hygiene

Bacterial strains	Plant extracts and non-botanical oral hygiene products, average inhibition zone (mm^2^)							Standard antibiotic
	Turmeric (*Curcuma longa*)	Miswak (*Salvadora persica*)	Derum (*Juglans regia L. bark*)	Charcoal	Sodium bicarbonate	Aloedent Anti-Cavity Sensitive toothpaste	Mouthwash	Erythromycin (15 µg)
Gram-positive								
*S. mutans*	8 ± 0	8 ± 0	16 ± 0	8 ± 0	8 ± 0	8 ± 0	8 ± 0	28±1.3
*S. aureus*	8 ± 0	8 ± 0	14.3 ± 2.08	8 ± 0	8 ± 0	14 ± 0	14 ± 0.57	24 ± 1.41
Gram-negative								
*E. coli*	8 ± 0	8 ± 0	14 ± 0	8 ± 0	8 ± 0	8 ± 0	12 ± 0.76	6 ± 0


**Fig 1:**
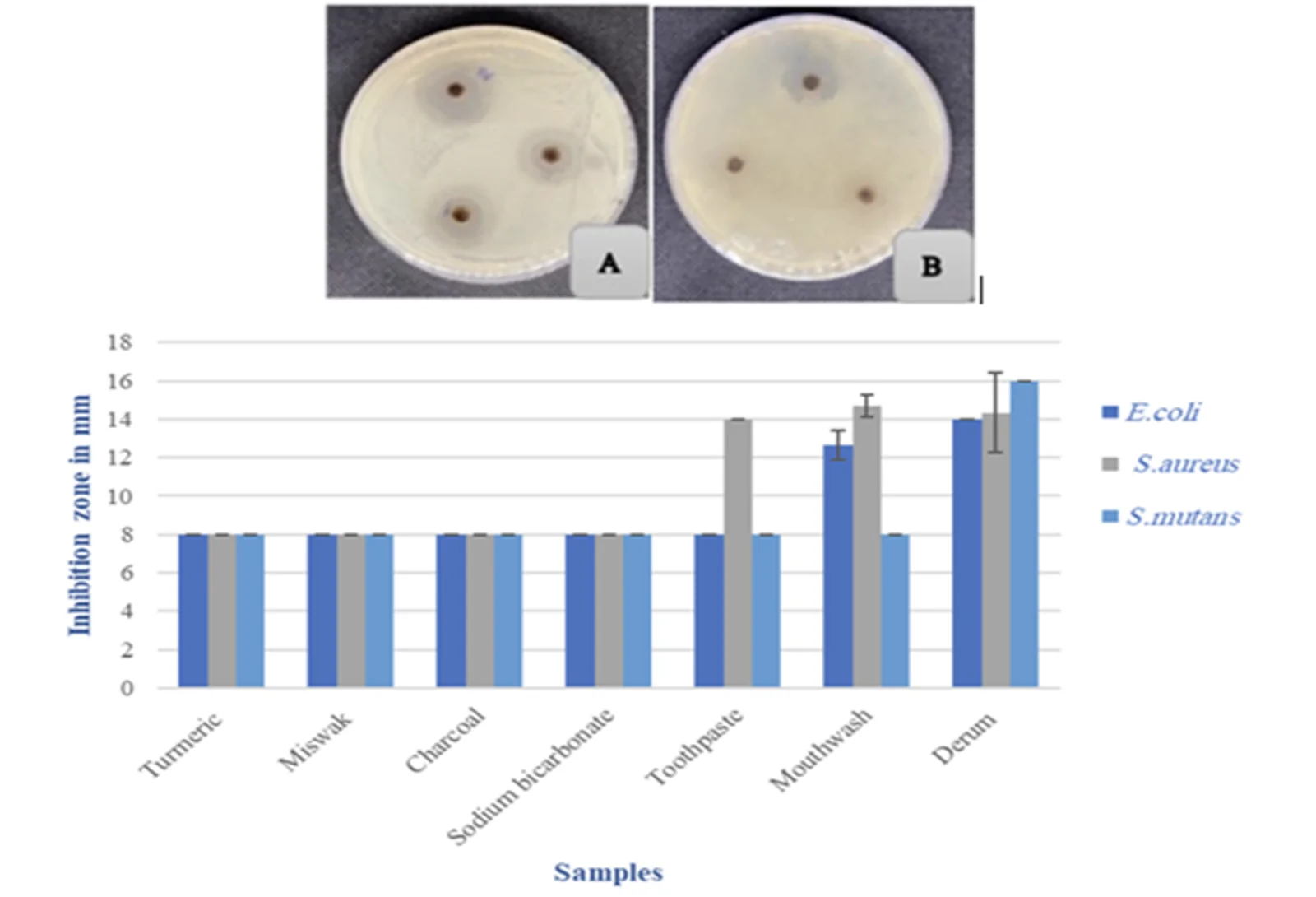
Derum extract’s antibacterial effect on A) *S. mutans* and B) *S. aureus* observed as inhibition halos around the respective wells on the agar surface, and the antibacterial activities of the various extracts and non-botanical oral-hygiene products. The highest activity was observed with derum on *S. mutans*.

### LC-MS/MS Detection

The LC–MS chromatogram revealed a small peak at m/z 279, corresponding to the deprotonated molecular ion ([M–H^⁻^]) of linoleic acid, indicating its presence in the derum extract. In addition, a prominent ion at m/z 265 was detected, which is consistent with an oxidized or fragmented derivative of linoleic acid and has been reported as a characteristic fingerprint ion of linoleic acid during mass spectrometric analysis. Furthermore, fragment ions at m/z 135, 159, 175, and 293 were also observed, which could be diagnostic ions of oxidized linoleic acid, providing additional evidence for the presence of linoleic acid in the extract (Fig 2). The detection of LA oxidative metabolites could be related to the preparation of the derum extract in water, where LA mainly loses its stability and decomposes into oxidized compounds.

**Fig 2:**
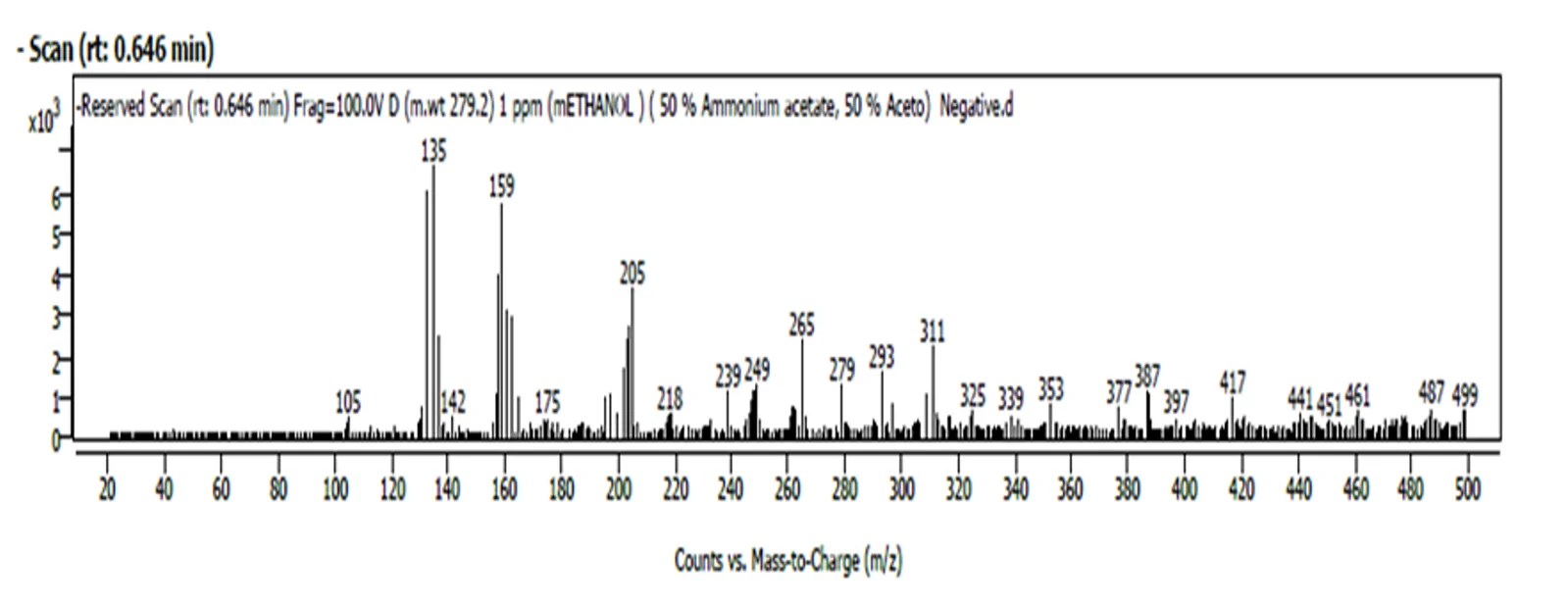
Derum extract LC-MS chromatogram showing the linoleic acid at 279m/z, in addition to the characteristic fingerprint of oxidized linoleic acid at 265m/z.

### Structure Prediction

Based on the LC-MS analysis, linoleic acid was identified as the polyunsaturated fatty acid present in the derum extract. Linoleic acid 2D and 3D structures were prepared and optimized using Chemsketch as shown in Fig 3A. Alpha-1,3-glucan was visualized using the Biovia Discovery studio visualizer (Fig 3B).

**Fig 3:**
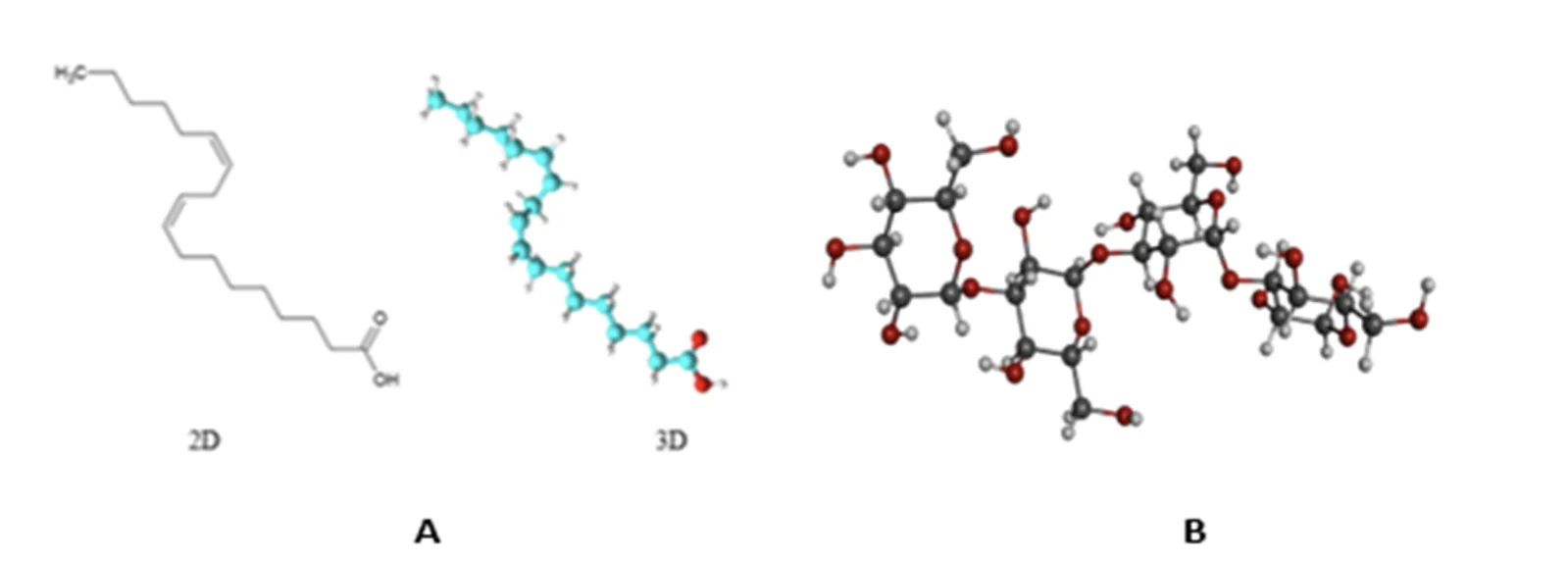
Predicted structures of A) linoleic acid and B) alpha 1,3-glucan retrieved using the Biovia Discovery Studio visualizer.

### Linoleic Acid Molinspiration Results 

Predictions of linoleic acid bioactivity were inferred from the findings of the set default parameters with predicted scores from molinspiration (Table 2). Linoleic acid presented a higher miLogP value, suggesting the compound’s lipophilic nature.

**Table 2:** Physiochemical characteristics of linoleic acid

Property name	Predicted value	2D	3D
miLogP	6.86	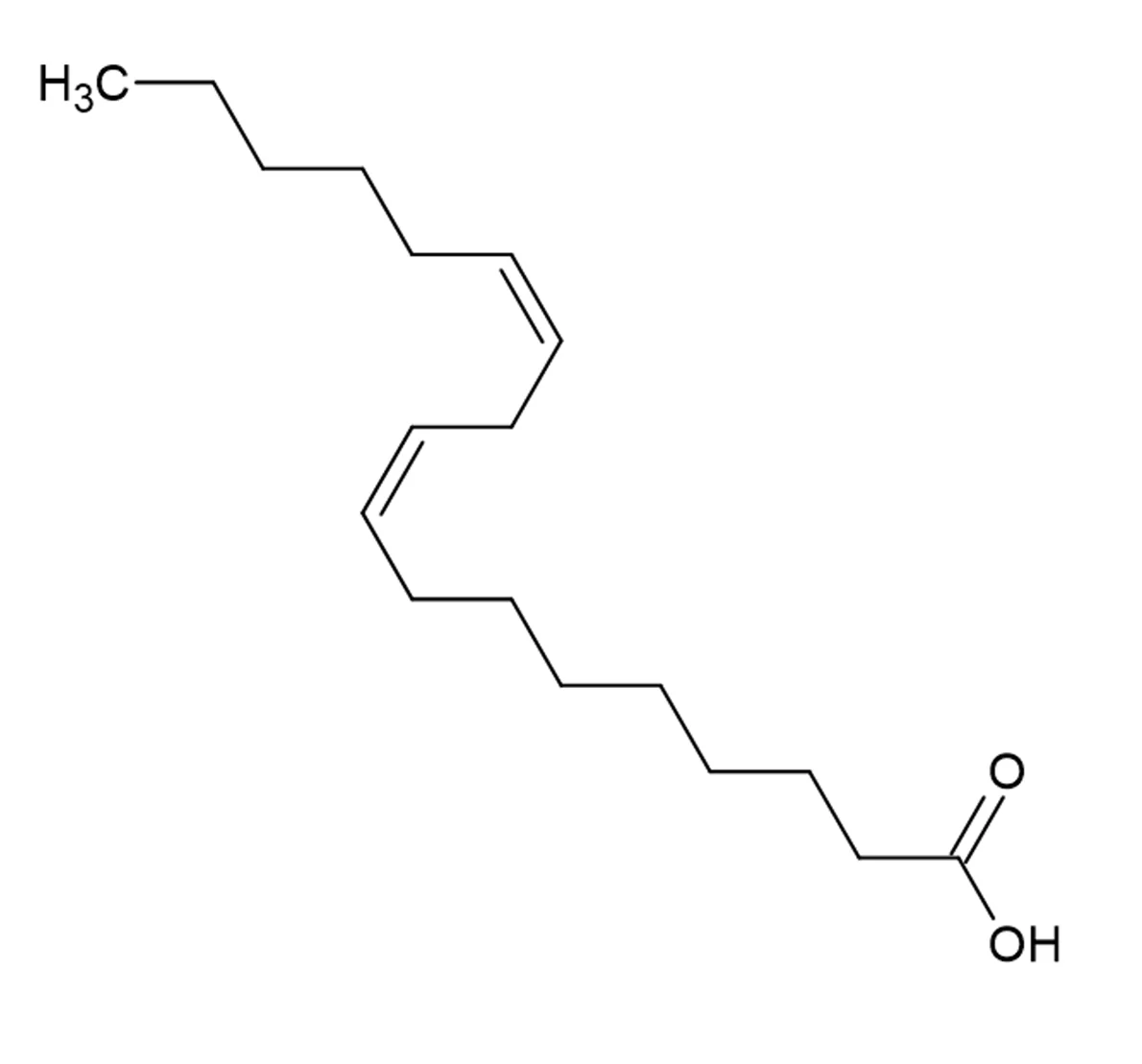	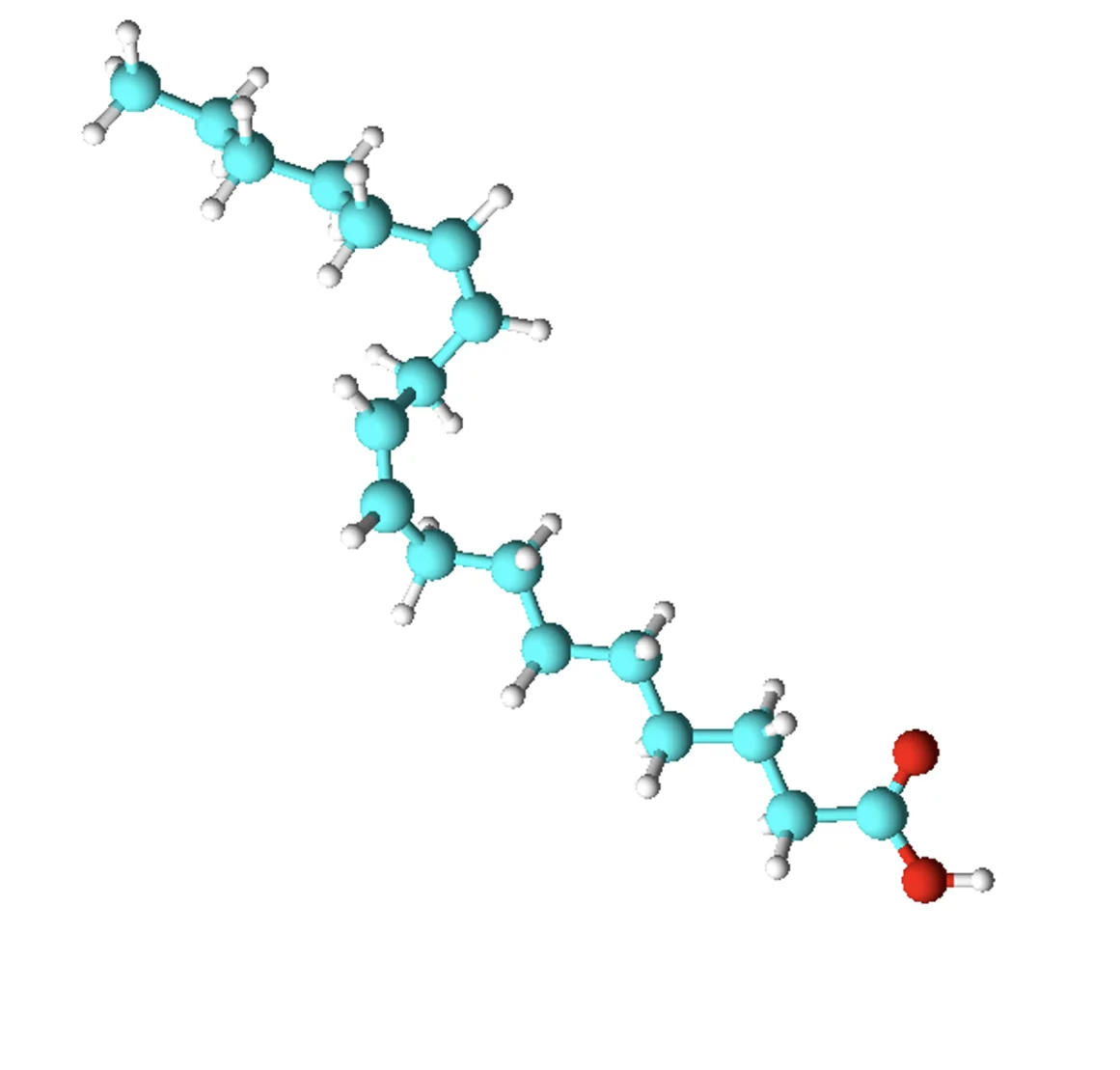
TPSA	37.30		
natoms	20		
MW	280.45		
nON	2		
nOHNH	1		
nrotb	14		
Volume	312.65		


### In-vitro Docking Analysis

Alpha 1,3-glucan is known to be present in the biofilm formed by *S. mutans*, thus screening the ability of linoleic acid (LA) to block and prevent the colonization and adhesion of *S. mutans,* and thus consequently biofilm formation, using docking simulation was the study aim along with the revealed antibacterial activity.

Based on the extracellular alpha-glucan matrix/linoleic acid conformations, the most suitable conformers were chosen with the aid of the Lamarckian Genetic Algorithm (LGA) based on binding energy. The least energy conformation was chosen with the binding energy of -1.24 kcal/mol. The ball-and-stick models of hydrogen bond interactions in linoleic acid against the alpha-1,3 glucan were visualized using the Biovia Discovery Studio visualizer. The conformer of alpha-1,3 glucan formed four hydrogen bonds with linoleic (Fig 4). The number of hydrogen bonds formed in concert with the torsional energy and the scores after docking between the target and linoleic acid are given in Table 3. The strong hydrogen bonds (1.84 Å and 3.01 Å) indicated that the fatty acid (LA) mimics the *S. mutans* active polymer sites; it binds directly to the EPS enzyme sites and alters the matrix.

**Fig 4:**
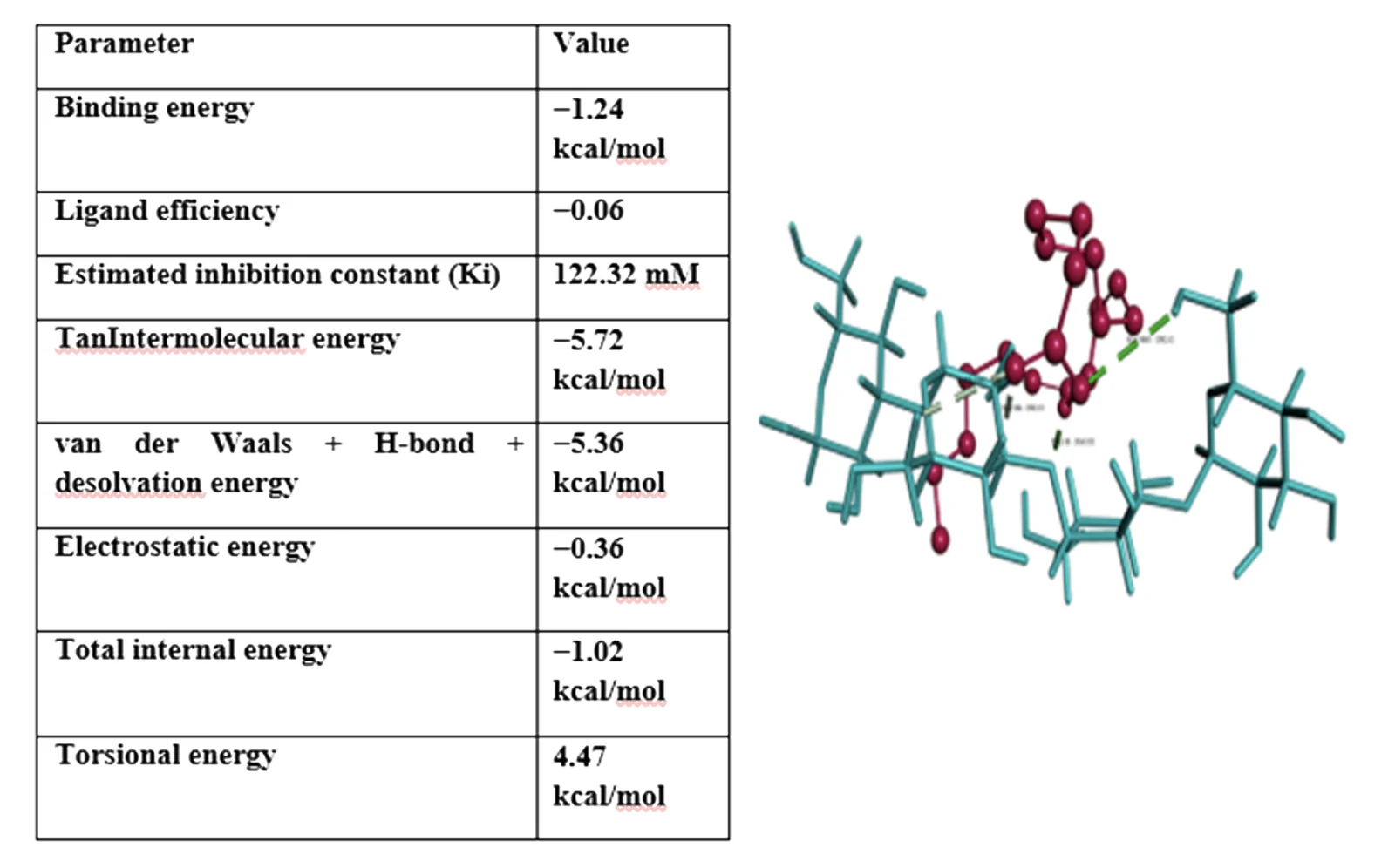
Energy binding confirmation and affinity interaction simulation between linoleic acid and alpha 1,3-glucan (scaled ball-and-stick in pink: linoleic acid; cyan-blue-colored stick-model: beta-glucan) as visualized using Biovia Discovery Studio Visualizer depicting the presence of four hydrogen bonds as dashed green lines between beta- glucan and linoleic acid.

**Table 3:** Alpha-1,3-glucan and linoleic acid hydrogen bond interactions

Interaction	Type	Distance (Å)
0GA5:H6O – UNL1:O	Conventional hydrogen bond	3.01
UNL1:H – 3GA3:O3	Conventional hydrogen bond	1.84
3GA3:H4 – UNL1:O	Carbon–hydrogen bond	1.84
3GA3:H61 – UNL1:O	Carbon–hydrogen bond	2.73


Higher affinity was observed between the ligand linoleic acid (LA) and alpha-glucan extracellular matrix based on low energy levels of -1.24 kcal/mol, as indicated by the conformation map and binding energy with the aid of the LGA (Fig 4). The lower the binding score, the higher the affinity is, and the thicker the link, the stronger the relationship is. The number of hydrogen bonds formed in concert with the torsional energy and the scores after the docking between the target and linoleic acid are also shown in Fig 4.

Four dominant hydrogen bonds were noted between linoleic acid and α-glucan (Fig 4). The ball-and-stick models of hydrogen bond interactions in linoleic acid against the alpha-glucan (cyan-blue stick model) were visualized using the Biovia Discovery studio visualizer. The distances of four hydrogen bonds formed between the oxygen atoms of linoleic acid and the hydroxyl groups of α-1,3-glucan were 1.84 Å to 3.01 Å (Table 3), indicating that the docking procedure was successfully validated and the interaction was strong.

## DISCUSSION 

Many conventional antimicrobial products are utilized to prevent dental plaque formation and inhibit the growth of cariogenic oral pathogens. However, they have only short-term efficacy and bear a high risk of increasing antibacterial resistance, among other relevant disadvantages. Therefore, there is a global demand for novel plant-based (“natural”) agents that are able to reduce cariogenic biofilm formation without negatively affecting the physiological oral microbial flora.

Throughout history, medicinal plants have been extensively used to promote health, and in modern times their antimicrobial activity has been extensively documented.^4^ The World Health Organization (WHO)^33^ postulated that chewing sticks (e.g., miswak, derum) are powerful oral hygiene tools, as they are widely available, potent, inexpensive, and have many therapeutic properties. Chewing sticks help mechanically clean the teeth by removing dental plaque and food remnants, and chemically by their known antibacterial constituents, thus fostering good oral hygiene.^11^

In this study, the antimicrobial effects of different plant extracts (turmeric, miswak, and derum) and non-botanical oral. and dental-care products (charcoal toothpaste, aloe-vera toothpaste, mouthwash, and sodium bicarbonate) were investigated on three bacterial species *Streptococcus mutans, Staphylococcus aureus, *and* Escherichia coli* which are the main pathogens responsible for dental caries. Our data indicate that most of the tested non-botanical substances and phyto-extracts did not show potent antibacterial activity, except for the derum sticks, which demonstrated the highest antibacterial activity against *S. mutans* (DIZ 16 mm^2^) (Table 1 and Fig 1), followed by *S. aureus* (DIZ 14.3 mm^2^) and *E. coli* (DIZ 14 mm^2^). This finding was consistent with previous studies^4,27^ which indicated that derum’s antibacterial effect can be impaired by several chemicals, such as phenolics and polyphenolics. Derum can increase salivary pH and consequently can affect bacterial growth.^5^ Polyphenols such as flavonoids, lignans, and tannins are not the only antimicrobial found in the bark of *Juglans regia*. Juglon, glycosides, and the polyunsaturated fatty acid LA (Fig 2) are also among the strongest antimicrobials in the bark of *Juglans regia*,^15^ making derum sticks the highly effective plaque-preventive tools in addition to their ease of use and availability.^15^ The presence of LA was indicated by the appearance of a small peak at m/z 279 in the LC–MS chromatogram (Fig 2), as was also detected by Kim et al.^17^ On the other hand, the mouthwash, charcoal toothpaste, aloe-vera toothpaste, and sodium bicarbonate revealed weak or negligible activity against the three tested bacterial species (DIZ 8 mm^2^). Among the aforementioned materials, the mouthwash exhibited the highest inhibitory activity on *S. aureus* (14 mm) and *E. coli* (12 mm), but no or negligible effect was noted on *S. mutans* (DIZ 8 mm^2^). Similarly, the Aloedent Anti-Cavity Sensitive toothpaste demonstrated the highest activity on *S. aureus* (DIZ 14 mm^2^) but did not significantly affect *S. mutans* or *E. coli* (DIZ 8 mm^2^), suggesting that adding derum to non-botanical dental hygiene products may prove beneficial. This variation in the results could be due to the resistance of the bacterial strains tested and/or to the non-botanical commercial dental hygiene products used, again pointing to the importance of integrating derum extracts into a variety of commercial oral-care products.

Nonetheless, *S. mutans* glucosyltransferases (Gtfs) play an essential role in pathogenesis of dental caries. Gtfs catalyse alpha 1,3-glucan formsfrom sucrose in the biofilm matrix via transglucosylation reactions; a drug that mimics and blocks the Gtfs active sites would be required to prevent caries. Therefore, the molecular docking simulation in the present studywas performed to demonstrate the ability of linoleic acid to block alpha glucan sites, preventing biofilm matrix formation,^34^ or making it more fragile, permeable, and highly susceptible to anti-biofilm agents. The molecular docking simulation of linoleic acid against the extracellular glucan matrix model yielded a binding energy of -1.24 kcal/mol (Fig 4). While this represents a weak thermodynamic affinity compared to classic drug-receptor interactions, it characterizes a highly dynamic and reversible surface adsorption mechanism. This weak affinity, together with a stabilized structure of four hydrogen bonds formed between the oxygen atoms of linoleic acid and the hydroxyl groups of α-1,3-glucan with distances from 1.84 Å to 3.01 Å (Table 3) contribute to ligand stabilization by acting as a solvent bridge within the binding region. The interaction pattern indicated that the carboxyl oxygen of linoleic acid serves as the principal hydrogen acceptor, whereas the abundant hydroxyl groups of α-1,3-glucan function as hydrogen donors. This is consistent with the highly hydrophilic nature of polysaccharides and the polar head group of linoleic acid. The hydrophobic hydrocarbon chain of linoleic acid contributes minimally to specific matrix interactions, explaining the low net binding affinity. Taken together, these data suggest that phyto-constituents from *Juglans regia* are not permanently trapped within the biofilm matrix. Instead, they act as transient surface-active agents (surfactants), lowering the cohesive integrity of the EPS, and facilitating the detachment of *S. mutans* plaque from the enamel surface. Therefore, when plant extracts and/or traditional chewing sticks such as derum are used in the oral cavity, the linoleic acid plays a dual role in combating dental caries and promoting oral health.

## CONCLUSION 

In terms of inhibiting dental plaque and affecting the growth rate of cariogenic microbes, derum sticks were found to possess the highest antibacterial activity against *S. mutans*, followed by *S. aureus* and *E. coli*. The mouthwash and toothpaste samples both were effective against *S. aureus* and *E. coli,* respectively, but not against *S. mutans,* charcoal, or sodium bicarbonate. Moreover, the insights from docking studies and biofilm disruption strategies can lead to the development of therapeutic agents targeting EPS synthesis.

### AI Statement

The assistance of artificial intelligence (AI) was not used in the manuscript.

## REFERENCES
